# Plasma Osteopontin Levels and Adverse Cardiovascular Outcomes in the PEACE Trial

**DOI:** 10.1371/journal.pone.0156965

**Published:** 2016-06-10

**Authors:** Ahmed D. Abdalrhim, Tariq S. Marroush, Erin E. Austin, Bernard J. Gersh, Nusret Solak, Syed A. Rizvi, Kent R. Bailey, Iftikhar J. Kullo

**Affiliations:** 1 Department of Cardiovascular Diseases, Mayo Clinic, Rochester, Minnesota, United States of America; 2 Department of Internal Medicine, Mayo Clinic, Rochester, Minnesota, United States of America; 3 Division of Biomedical Statistics and Informatics, Mayo Clinic, Rochester, Minnesota, United States of America; University of Glasgow, UNITED KINGDOM

## Abstract

Osteopontin (OPN) is a secreted glycophosphoprotein that has a role in inflammation, immune response and calcification. We hypothesized that plasma OPN levels are associated with adverse cardiovascular outcomes in patients with stable coronary artery disease (CAD) and preserved ejection fraction (EF) enrolled in the PEACE trial. We measured plasma OPN levels at baseline in 3567 CAD patients (mean age 64.5 ± 8.1 years, 81% men) by a sandwich chemiluminescent assay (coefficient of variation = 4.1%). OPN levels were natural log (Ln) transformed prior to analyses. We assessed whether Ln OPN levels were associated with the composite primary endpoint of cardiovascular death, non-fatal myocardial infarction and hospitalization for heart failure using multiple event multivariable Cox proportional hazards regression. Adjustment was performed for: (a) age and sex; (b) additional potential confounders; and (c) a parsimonious set of statistically significant 10 variates. During a median follow-up of 4.8 years, 416 adverse cardiovascular outcomes occurred in 366 patients. Ln OPN was significantly associated with the primary endpoint; HR (95% CI) = 1.56 (1.27, 1.92); *P* <0.001, and remained significant after adjustment for age and sex [1.31 (1.06, 1.61); *P* = 0.01] and after adjustment for relevant covariates [1.24 (1.01, 1.52); *P* = 0.04]. In a secondary analysis of the individual event types, Ln OPN was significantly associated with incident hospitalization for heart failure: HR (95% CI) = 2.04 (1.44, 2.89); *P* <0.001, even after adjustment for age, sex and additional relevant covariates. In conclusion, in patients with stable CAD and preserved EF on optimal medical therapy, plasma OPN levels were independently associated with the composite incident endpoint of adverse cardiovascular outcomes as well as incident hospitalization for heart failure.

## Introduction

Coronary heart disease remains the leading cause of death in the U.S. [1,2], and affected patients are at risk for recurrent adverse cardiovascular outcomes. Consequently, there is great interest in the use of biomarkers to identify patients at increased risk of subsequent cardiovascular events who may benefit from aggressive treatment. Osteopontin (OPN), an acidic glycoprotein [3,4,5] involved in immune-inflammatory processes and normal and pathological calcification [3,6], is highly expressed in calcified atherosclerotic plaques, valves, and neointimal smooth muscle cells [7,8]. OPN levels are higher in symptomatic carotid atheromas than in asymptomatic atheromas [9], and are associated with the presence and extent of coronary atherosclerosis on coronary angiography [10,11,12]. Furthermore, it is speculated that OPN may influence atherosclerotic plaque formation, instability and rupture [13]. OPN’s role in calcification has a role in increased vascular resistance and hence the development of heart failure.

Despite the implication of OPN in pathways relevant to atherosclerosis, few studies have investigated whether levels of OPN are associated with adverse cardiovascular outcomes in patients with known cardiovascular disease [14,15,16]. In two prior studies of patients with stable coronary artery disease (CAD), higher OPN plasma levels were associated with adverse cardiovascular outcomes [14,16]. In another study, OPN levels were associated with adverse cardiovascular outcomes in patients with ST-elevation myocardial infarction (MI) who underwent primary percutaneous coronary intervention [15]. However, no previous study has investigated the association of OPN with adverse cardiovascular outcomes in patients with stable CAD who are on optimal medical treatment. Therefore, we assessed the prognostic impact of plasma levels of OPN at baseline in patients with stable CAD and preserved left ventricular ejection fraction (EF), and who are on optimal medical treatment. We hypothesized that increased OPN levels would be associated with adverse cardiovascular outcomes: cardiovascular death, non-fatal myocardial infarction and hospitalization for heart failure. Our primary endpoint was a composite of these three events, but each event was individually studied (secondary hypotheses).

## Methods

### Measurement of OPN levels

This research was approved by the Mayo IRB. Participant records/information was anonymized and de-identified prior to analysis. We measured plasma OPN levels at baseline in 3567 patients who constitute a subset of the two arms of the PEACE trial [17]. Eligible patients had stable CAD with an EF >40%. Stable CAD was defined as either a history of myocardial infarction (MI) at least three months before enrollment, a history of coronary artery bypass grafting (CABG) or percutaneous transluminal coronary angioplasty (PTCA) at least three months before enrollment, or more than 50% narrowing of one of the coronary arteries on angiography. Further details about inclusion and exclusion criteria have been previously reported [17]. Patients were on optimal medical treatment and were randomized to Trandolapril or placebo arms between November 1996 and June 2000 and followed at six-month intervals for up to 7 years (median follow-up, 4.8 years), until December 31, 2003. We measured baseline OPN levels using a validated sandwich chemiluminescent assay on the MesoScale Discovery platform (MSD; Gaithersburg, MD). The lower limit of quantitation was 0.064 ng/mL, the upper limit of quantitation was 37.5 ng/mL, and the inter-assay coefficient of variation was 4.1%. The PEACE trial team used a World Health Organization certified methodology with respect to treatment of biomarkers. Further details about the OPN assay are included in the S1 Appendix.

### Statistical analysis

Analyses were performed using R, version 2.14.1 [18]. First, in order to account for the right-skewed distribution of OPN levels, we used natural logarithm transformed OPN (Ln OPN) in all analyses. Participants were at risk for multiple adverse events. We a priori focused on three types of events, which we aggregated into a composite outcome: cardiovascular death, MI and hospitalization for heart failure; that is, in our study a trial participant could have from zero to three recorded events (one each for the three components of the composite outcome). Thus, we did an initial exploratory analysis to assess the association between a patient’s Ln OPN level and the total number of adverse outcomes she or he experienced. This assessment was done to confirm the value of integrating this information into the survival modeling approach. Mean Ln OPN was calculated within groups defined by the number of adverse events incurred (0, 1, 2, or 3). Unadjusted p-values from two-sample t-tests of these group means were estimated because any significant differences in mean Ln OPN between the 1, 2, and 3 event groups would strongly necessitate using a multiple events survival model approach.

Kaplan–Meier (K-M) cumulative survival plots for both the first event of the composite endpoint and for individual event types were generated according to tertiles of Ln OPN. The plots are intended to substantiate a biomarker effect, one warranting detailed analysis of these prespecified adverse outcomes. Baseline sample characteristics were summarized at both the individual and Ln OPN tertile levels. As with the K-M plots, tertile groupings were used to provide a quick insight into the effect of increasing plasma levels. Consequently, *P*-values for trend were calculated using, as appropriate, either general linear hypothesis testing or the χ^2^ test for trend in proportions. We used multivariable linear regression analysis to identify variables associated with Ln OPN for both informative purposes and to ensure possible confounders were included in our modeling strategy. We reported the parsimonious model identified via a backwards stepwise methodology with a 0.05 inclusion threshold.

For the time-to-event analysis, Cox proportional hazards models (Cox PHMs) were first used to estimate the effect of the tertile of Ln OPN plasma levels on the endpoints for our primary and secondary hypotheses. This partitioned version of Ln OPN was utilized to confirm the related tertile Kaplan-Meier findings. Next, Cox PHMs were fit using the continuous form of Ln OPN plasma levels in order to directly test the study hypotheses. Four models were fit for each outcome: in model (1) we assessed the effect of Ln OPN on the endpoint without adjustment; in model (2) we adjusted for age and sex; in model (3) we adjusted for the full set of potential confounders including age, sex, treatment allocation (Trandolapril vs. placebo), weight, smoking status, treatment with lipid-lowering drugs, use of beta blockers, use of antiplatelet agents, left ventricular EF, systolic blood pressure, diastolic blood pressure, estimated glomerular filtration rate, and history of any of the following: diabetes, hypertension, angina, intermittent claudication, transient ischemic attack, stroke, MI, PTCA, and CABG; and in model (4) we use a backwards stepwise methodology to reduce model (3) to a parsimonious list of variables significant at the 0.05 level. Please note, model (4) is what is described as the parsimonious model in the following results and discussion sections. Multiple events were accounted for by calculating a unique baseline hazard for each of the three adverse outcomes and using robust sandwich variance estimators to account for within sample correlation. Death was treated as a censoring event in all models. Event time ties were handled with the Efron method. Hazard ratios (HRs) and corresponding 95% confidence intervals (CIs) are provided in addition to *P*-values based on Z-tests. The proportional hazards assumption was checked using the methodology of Grambsch and Therneau [19]. Last, relative Integrated Discrimination Improvement (rIDI), continuous Net Reclassification Improvement (NRI), and C-statistic comparisons (presented as relative improvement) of the adjusted models were generated to provide insight on Ln OPN's impact on discrimination.

## Results

**Fig 1:**
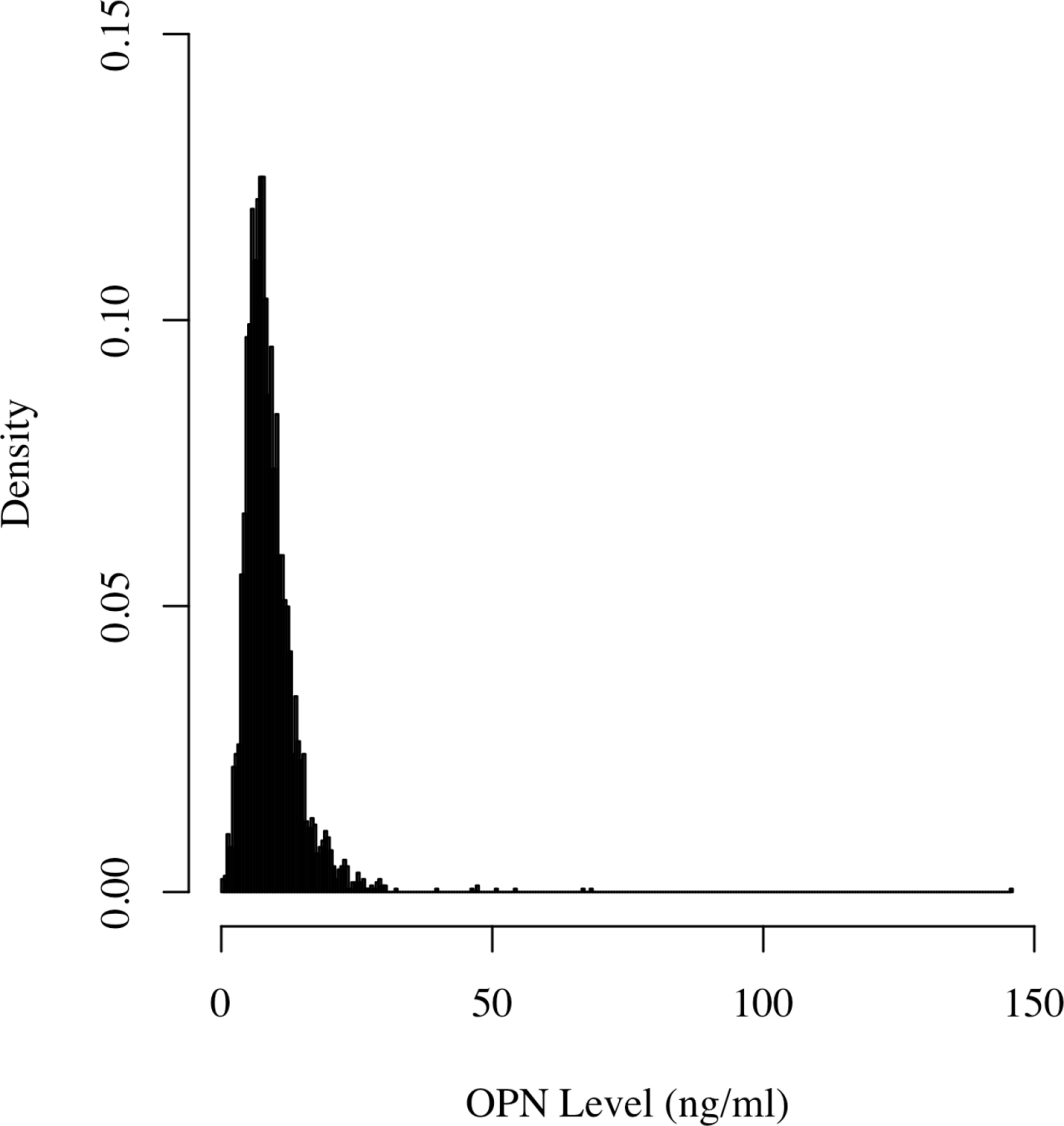
Histogram of OPN levels in 3567 participants.

The study cohort consisted of 3567 patients, the majority (81%) of whom were men and the mean (standard deviation) age was 64.5 ± 8.1 years. The median OPN level was 7.95 ng/ml with an interquartile range of 5.90 to 10.85 ng/ml. A distribution of OPN levels is given in Fig 1, revealing a severe skewness that we addressed by applying the well-established natural logarithm transformation to the OPN values. Patient characteristics are summarized in Table 1. As described previously, summaries are provided for both samples within the three Ln OPN tertiles and for the full combined group. The statistical significance of a large number of the trend tests further matched our a priori decision to adjust for a diverse covariate set in order to best minimize the effect of potential confounders on our OPN conclusions. Greater male sex, age, smoking, and history of stroke, CABG, and intermittent claudication were associated with higher Ln OPN levels, whereas greater weight, eGFR, history of diabetes, use of lipid-lowering and use of beta blocking drugs were associated with lower levels (Table 2). The association with age was remarkably strong (*P* <0.001), a finding also reported in the Framingham offspring cohort [20].

**Table 1:** Baseline Characteristics of the Study Patients.*

Variable	Combined	Tertile I	Tertile II	Tertile III	P
Osteopontin Range, ng/ml	[0.17,145.77]	[0.17,6.61]	[6.62,9.72]	[9.73,145.77]	-
Osteopontin, ng/ml	9.0 ± 5.4	4.9 ± 1.3	8.0 ± 0.9	14.1 ± 6.4	<0.001
Ln Osteopontin	2.1 ± 0.5	1.5 ± 0.4	2.1 ± 0.1	2.6 ± 0.3	<0.001
Men	2872 (81%)	929 (78%)	975 (82%)	968 (82%)	0.03
Age, years	64.5 ± 8.1	62.3 ± 7.7	64.2 ± 7.8	66.9 ± 8.0	<0.001
Weight, kg	83.8 ± 15.7	84.9 ± 15.8	84.6 ± 15.7	81.9 ± 15.4	<0.001
Smoking	2703 (76%)	879 (74%)	931 (78%)	893 (75%)	0.46
History of diabetes	589 (17%)	224 (19%)	179 (15%)	186 (16%)	0.04
Systolic blood pressure, mm Hg	133.5 ± 16.9	132.5 ± 16.6	133.2 ± 16.6	134.7 ± 17.4	0.002
Diastolic blood pressure, mm Hg	78.0 ± 10.0	78.6 ± 9.8	78.1 ± 10.0	77.4 ± 10.2	0.002
History of hypertension	1595 (45%)	509 (43%)	529 (44%)	557 (47%)	0.04
eGFR, mL/min	77.8 ± 19.4	80.1 ± 19.7	78.8 ± 18.5	74.5 ± 19.6	<0.001
Left ventricular EF (%)	58.8 ± 9.6	58.8 ± 9.6	58.7 ± 9.7	58.8 ± 9.6	0.996
History of TIA	116 (3%)	24 (2%)	42 (4%)	50 (4%)	0.003
History of angina	2595 (73%)	869 (73%)	870 (73%)	856 (72%)	0.62
History of myocardial infarction	2010 (56%)	677 (57%)	684 (58%)	649 (55%)	0.28
History of stroke	151 (4%)	41 (3%)	46 (4%)	64 (5%)	0.02
History of CABG	1273 (36%)	355 (30%)	395 (33%)	523 (44%)	<0.001
History of PTCA	1623 (46%)	575 (48%)	551 (46%)	497 (42%)	0.002
History of intermittent claudication	317 (9%)	88 (7%)	98 (8%)	131 (11%)	0.002
Beta blocking drugs	2204 (62%)	788 (66%)	738 (62%)	678 (57%)	<0.001
Lipid-lowering drugs	2565 (72%)	892 (75%)	868 (73%)	805 (68%)	<0.001
Trandolapril allocation	1775 (50%)	600 (50%)	596 (50%)	579 (49%)	0.42
Aspirin & Antiplatelet	3248 (91%)	1085 (91%)	1090 (92%)	1073 (90%)	0.05

* Mean ± Standard Deviation for continuous variables; count (percent of samples) for categorical variables. Abbreviations: CABG, coronary artery bypass grafting; eGFR, estimated glomerular filtration rate; EF, ejection fraction; PTCA, percutaneous transluminal coronary angioblasty; TIA, transient ischemic attack.

**Table 2:** Variables Associated with Ln OPN.

**Variable**	**β (95% CI)**	***P***
Male	0.119 (0.075, 0.164)	<0.001
Age, years	0.013 (0.010, 0.015)	<0.001
Weight, kg	-0.001 (-0.002, 0.000)	0.03
Smoking	0.042 (0.003, 0.081)	0.04
History of diabetes	-0.051 (-0.096, -0.007)	0.02
eGFR, mL/min	-0.002 (-0.003, -0.001)	<0.001
History of stroke	0.084 (0.002, 0.166)	0.04
History of CABG	0.096 (0.061, 0.130)	<0.001
History of intermittent claudication	0.068 (0.010, 0.126)	0.02
Beta-blocking drugs	-0.051 (-0.084, -0.017)	0.003
Lipid-lowering drugs	-0.053 (-0.090, -0.017)	0.004

Abbreviations: CABG, coronary artery bypass grafting; eGFR, estimated glomerular filtration rate.

**Fig 2:**
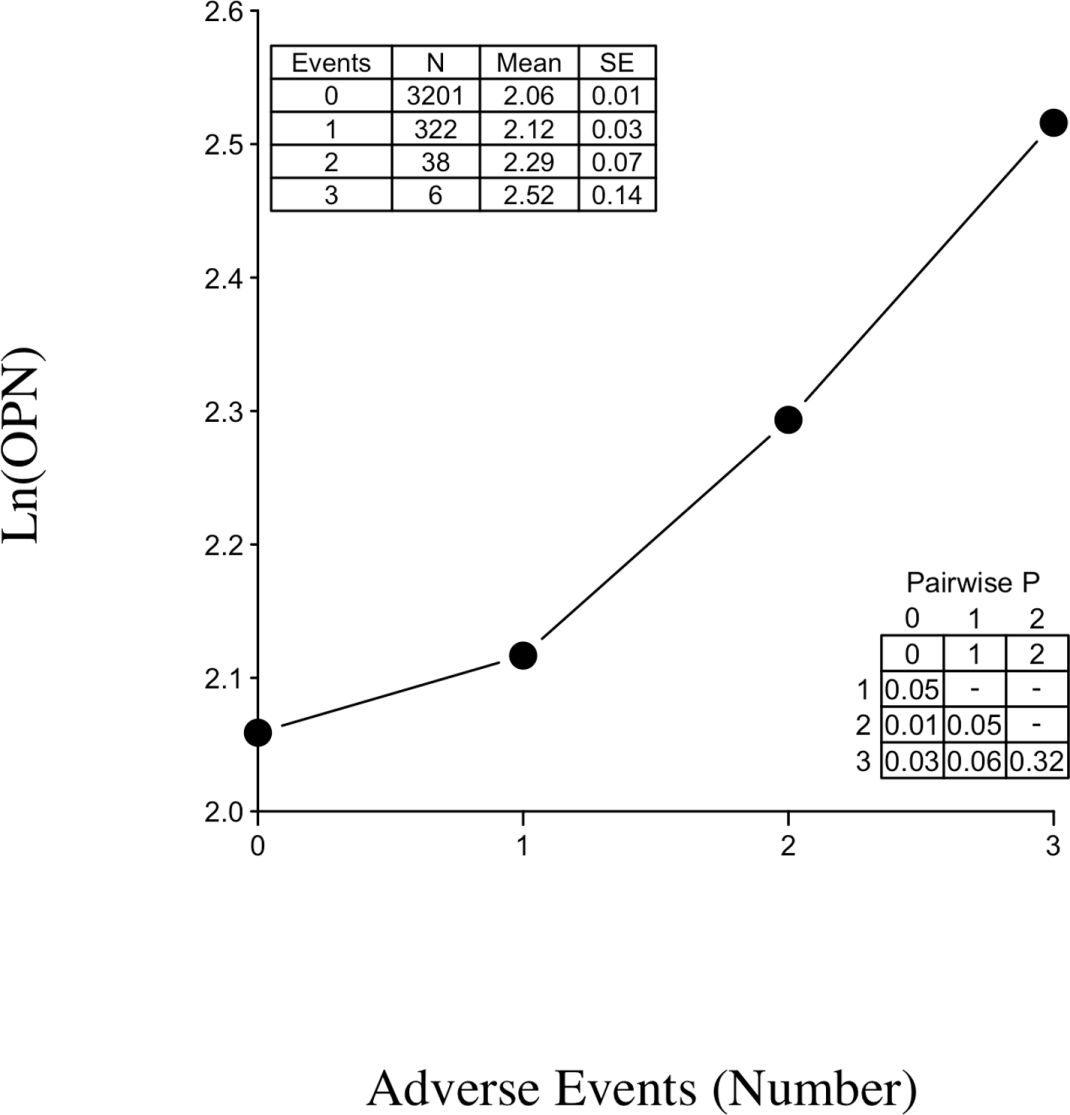
Scatterplot of mean Ln OPN level of samples grouped by number of adverse events incurred. Inset tables contain summary statistics and *P*-values for pairwise comparisons of the means.

At the end of the follow-up period, 416 adverse cardiovascular outcomes occurred (125 deaths from cardiovascular causes, 202 non-fatal myocardial infarctions and 89 hospitalizations due to heart failure). The primary endpoint was reached by 366 patients. The mean time to outcome was 1025 days for patients that had exactly one of the three recorded adverse outcomes. For those experiencing multiple outcomes, the first occurred on average at 837 days, with subsequent outcomes happening in approximately half the time (447 days after the first adverse event). Fig 2 plots the mean Ln OPN by number of incurred adverse events (grouping patients who experienced the same number of unique outcomes). Fig 2 also provides the sample sizes, means, and standard errors (SEs) in an inset table. As an example, for the 322 people who experienced exactly one of the three study cardiovascular events, their mean baseline Ln OPN was 2.12. There was a distinct increase in Ln OPN with an increased number of adverse events incurred by a patient. A second inset table in Fig 2 reveals a significant or near significant increase in those with two or three adverse events versus those experiencing only one of the three study outcomes (*P* = 0.05 and 0.06). Therefore, multiple event analysis was an integral feature of our survival analysis.

**Fig 3:**
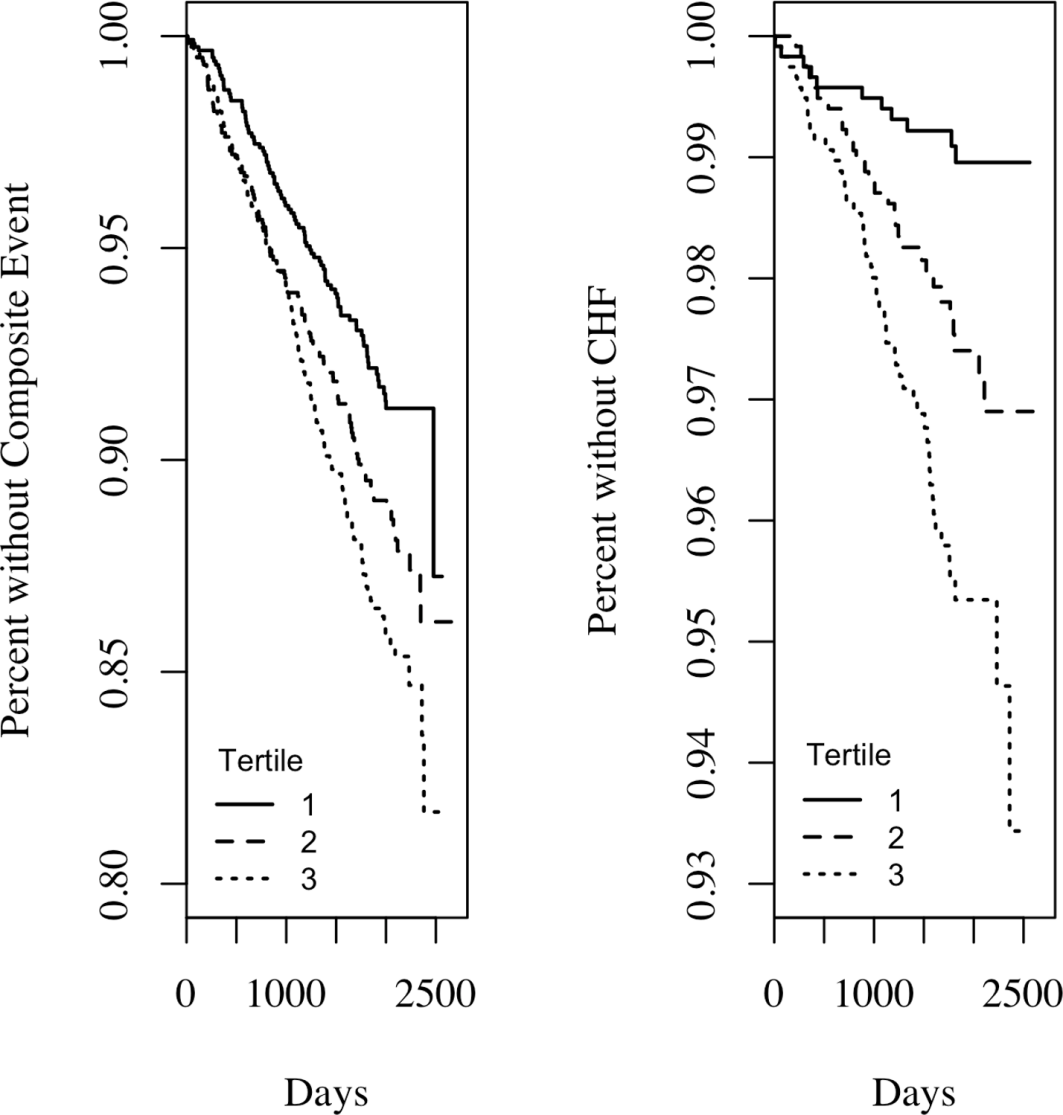
Kaplan–Meier survival plots. A) Kaplan–Meier survival plot for the composite of the adverse cardiovascular outcomes according to tertiles of Ln (OPN). Tertile I≤1.89, tertile II = 1.90–2.27, and tertile III ≥ 2.28. B) Kaplan–Meier survival plot for hospitalization for heart failure according to tertiles of Ln (OPN).

Kaplan-Meier curves depicting survival free of the composite endpoint and hospitalization for heart failure endpoint for tertiles of Ln OPN are shown in Fig 3(A) and 3(B). Survival free of adverse cardiovascular outcomes decreased by Ln OPN tertile (P <0.001). In the parsimonious Cox PHM, patients with Ln OPN levels in both the second tertile [HR (95% CI) = 1.40 (1.06, 1.85); P = 0.02)] and third tertile [HR (95% CI) = 1.50 (1.14, 1.97); P = 0.004)] had significantly greater risk of composite outcome than samples with a first tertile baseline Ln OPN level. Similarly, survival free of the hospitalization for heart failure endpoint was significantly associated with Ln OPN, decreasing with each increased tertile (P <0.001). Here, the parsimonious Cox PHM showed even more increased risk with both the second [HR (95% CI) = 2.52 (1.24, 5.09); P = 0.01)] and third [HR (95% CI) = 3.34 (1.70, 6.59); P = <0.001)] tertiles compared to tertile one.

**Fig 4:**
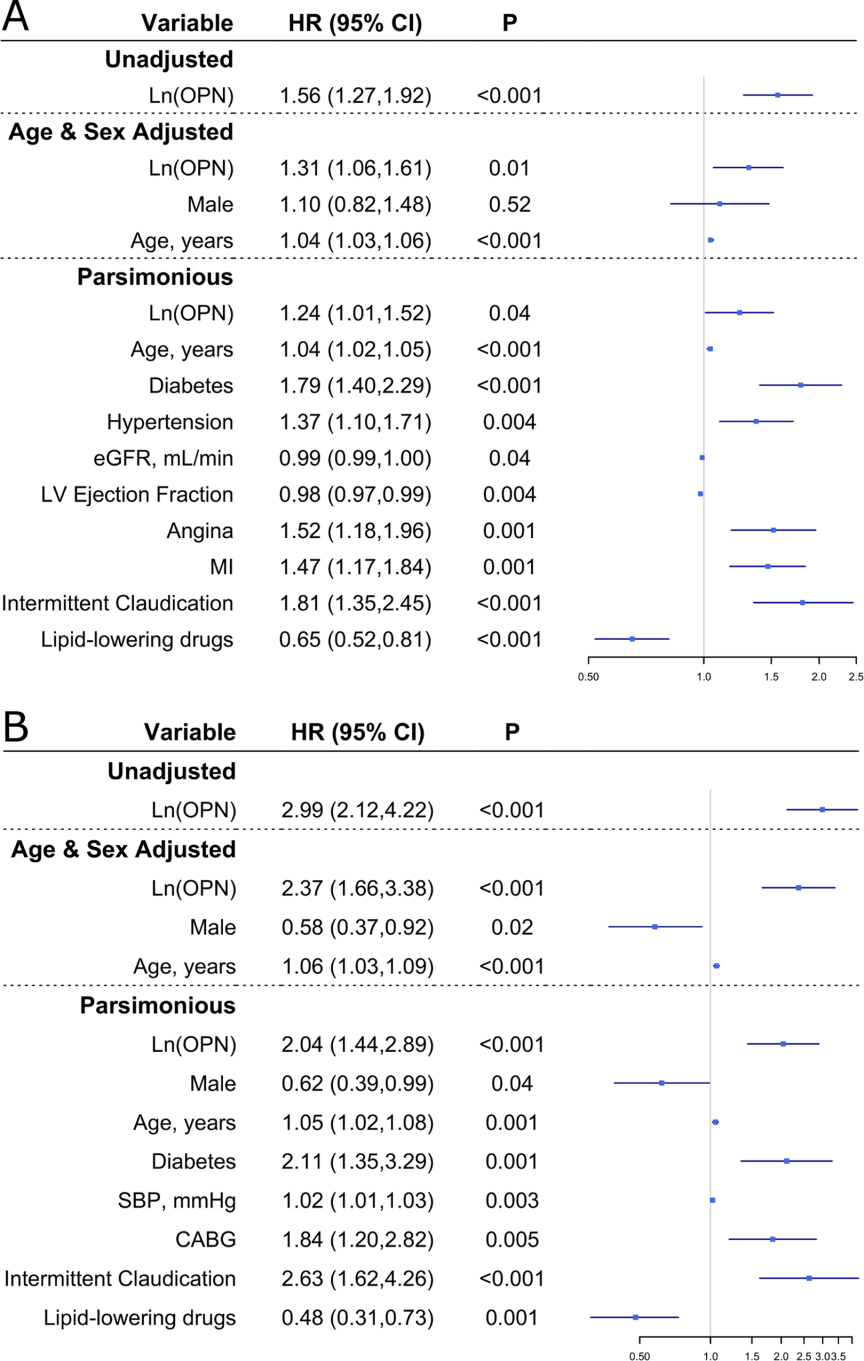
Cox Proportional Hazard Model results. A) Hazard Ratios (95% Confidence Intervals) and *P*-values for the composite of the adverse cardiovascular outcomes. B) Hazard Ratios (95% Confidence Intervals) and *P*-values for hospitalization for heart failure.

The analysis by tertile of Ln OPN showed a robust general effect, giving a credible framework for any potential continuous effect finding, which we assessed subsequently. In the main effect Cox proportional hazards regression model where we were treating the biomarker as continuous, a unit increase in Ln OPN was significantly associated with higher risk of reaching the composite endpoint [HR (95% CI) = 1.56 (1.27, 1.92); P <0.001)]. This association remained significant after adjustment for age and sex [HR (95% CI) = 1.31 (1.06, 1.61); P = 0.01)]. While Ln OPN was only approaching significance in the full model [HR (95% CI) = 1.20 (0.98, 1.48); P = 0.08)], it was highly noteworthy with respect to our primary hypothesis that Ln OPN’s association was statistically significant after removing the unassociated covariates [HR (95% CI) = 1.24 (1.01, 1.52); P = 0.04)]. These results are shown in Fig 4(A) for all but the full model. The four models including Ln OPN had significant concordances as assessed by Harrell’s *c*-indexes (SEs) of 0.57 (0.03), 0.61 (0.03), 0.70 (0.03), and 0.70 (0.03). For the age and sex adjusted Cox PHM, Ln OPN increased, but not to a statistically significant magnitude, the C-statistic (1.2%; P = 0.36) and had positive, but again not statistically significant, rIDI (0.54%; P = 0.35). Please remark how the continuous NRI achieved near significance (0.085; P = 0.06), providing further evidence of the value of OPN when estimating risk for the composite event. Ln OPN’s effect on discrimination was similar in the parsimonious model where the C-statistic increased 0.24% (P = 0.47), rIDI was 0.70% (P = 0.39), and continuous NRI was 0.068 (P = 0.11). An interaction term between Ln OPN and the treatment was insignificant (P = 0.37) and thus not considered in models 3 and 4. Ln OPN met the proportional hazards assumption in all models (P > 0.05).

When analyzing the associations of Ln OPN with individual event types (for the secondary hypotheses), Ln OPN was significantly associated with hospitalization due to heart failure in all models (*P* <0.001 in all models) (Fig 4(B)). In the parsimonious model the effect was quite strong as reflected in a hazard ratio (95% CI) of 2.04 (1.44, 2.89). Like the composite endpoint, the four models for hospitalization for heart failure had significant concordances as measured by Harrell’s *c*-indexes (SEs) of 0.66 (0.03), 0.71 (0.03), 0.81 (0.03), and 0.79 (0.03). For the age and sex adjusted Cox PHM, Ln OPN increased the C-statistic more than with the composite endpoint (6.25%) but still not significantly (P = 0.17). For this model both rIDI (42.12%; P = 0.01) and continuous NRI (0.334; P < 0.01) were significant. The effect on discrimination of Ln OPN was again similar in the parsimonious model where the C-statistic increased 1.29% (P = 0.41), rIDI was 19.88% and statistically significant (P = 0.02), and continuous NRI was 0.315 and likewise significant (P < 0.01). An interaction term approached significance (*P* = 0.06) in the full model, but the corresponding main effect of treatment was noticeably not associated in this modeling scenario (*P* = 0.16). Additionally, the significance of Ln OPN remained as strong (*P* <0.01). Because of this, the interaction term was not included in models 3 and 4. Ln OPN met the proportional hazards assumption in all models (P > 0.05). Associations between Ln OPN and either cardiovascular death or non-fatal myocardial infarction were not significant after any adjustment (results not shown).

## Discussion

In this study we demonstrate that in patients with stable CAD and preserved EF, baseline plasma OPN levels were significantly associated with incident adverse cardiovascular outcomes. Although OPN levels were strongly associated with age and sex, the association with the composite outcome remained significant after adjustment for these and additional potential confounder variables. When analyzing hospitalization due to heart failure separately, higher OPN levels were associated with greater risk, independent of age, sex, and other potential confounders.

OPN levels were strongly associated with age in our cohort, and a similar association was noticed in Framingham offspring study cohort [20]. Increased expression of bFGF, TGF/3 and angiotensin II with age [21], as well as age related osteoporosis and vascular calcification [20] may underlie this association. Levels were also strongly associated with sex, being higher in men than women. This may be due to estrogen-induced suppression of OPN expression in vascular smooth muscle [20]. Of note, statin use was significantly associated with lower plasma OPN levels. A previous report made a similar observation [22], indicating lower OPN levels in those on statin therapy.

In our cohort, OPN levels were similar in patients who experienced MI to those who did not. Levels were significantly higher in those who died due to cardiovascular pathologies; however, this was due to confounding covariates, particularly age. Minoretti et al. [14] demonstrated that higher levels of OPN are an independent predictor of adverse cardiovascular outcomes in patients with chronic stable angina. The endpoint analyzed in that study was a composite of cardiovascular death and non-fatal MI, whereas in our study, we also included heart failure in our analysis. However, no significant association was noted in our cohort when analyzing cardiovascular death and non-fatal MI as endpoints (analysis not shown). Several differences in the study cohorts may explain the different results including number of patients (3567 vs. 799), follow up duration (median 4.8 years vs. 2.7 years) [14] and use of lipid-lowering drugs (72% vs. 38%).

OPN has been studied in the context of heart failure [23,24,25,26]. Studies have shown that angiotensin II induces wound healing and cardiac remodeling by enhancing OPN expression in the injured myocardium [23], and that OPN is a marker of fibroblast and myofibroblast differentiation and maturation after cardiac injury [24]. Increased OPN expression was observed in the myocardium concurrent with the development of heart failure [27]. OPN has also been linked also to heart failure severity [25], and progression [26]. Although our findings suggest a potential role of OPN in heart failure, a causal relationship between OPN and heart failure is not yet established, and additional investigation is needed to examine a possible role of OPN in the pathogenesis of heart failure.

This study has several limitations: The majority of patients were Caucasian males, limiting the power to uniquely assess women and individuals of a non-European ancestry. Also, the measurements were done using samples provided at only baseline. Finally, patients were on ideal medical treatment for the most part, a state that is not representative of all patients with CAD.

We conclude that baseline plasma OPN levels are independently associated with the composite of cardiovascular death, non-fatal MI and hospitalization for heart failure in patients with stable CAD and preserved EF. Higher OPN levels were independently associated with higher incidence of heart failure hospitalization. Further work is needed to clarify a potential role of OPN as a biomarker for predicting development of heart failure in stable CAD patients with stable EF.

## Supporting Information

S1 Appendix Additional methods. Further details about the OPN assay.
(DOCX)
