# Supplementary material for: Plasma Osteopontin Levels and Adverse Cardiovascular Outcomes in the PEACE Trial
Source: PLoS One. 2016 Jun 10;11(6):e0156965. doi: 10.1371/journal.pone.0156965 (PMC4902195; doi:10.1371/journal.pone.0156965)
Supplement: S1 Appendix — Further details about the OPN assay. (DOCX) [file pone.0156965.s001.docx]

**S1 Appendix**

**S1. Additional methods**. Further details about the OPN assay.

At the time of enrollment in the PEACE trial, a blood sample was obtained by venipuncture from each participant in an EDTA-treated tube, samples were centrifuged and plasma was separated and frozen. Samples were transferred to our lab on dry ice where they were stored at -70° until the time of assay, then thawed them on ice or the day of OPN measurement. After blocking the plates overnight using the bovine A blocker, we thawed samples on ice, and diluted samples 20 fold using diluent 7. Calibration was done using the Human Bone Panel II Calibrator at a concentration of 0.2 µg/mL which was diluted sequentially using the diluent 7. We used diluent 7 alone as a blank calibrator. Samples were added as duplicates to the wells, and put on a shaker for two hours. Afterwards, wells were washed using the 1X PBS (phosphate buffer solution). To prepare the detection antibody we added 2.94 mL of diluent 11 to 60 µL of 50X SULFO-TAG Anti-hOsteopontin Antibody (final concentration: 1X), then we added 25 µL of the antibody solution to each well, we incubated for one hour on a shaker at room temperature. After that, the wells were washed using the PBS 1X, and 150 µL of the 1X MSD Read buffer T solution were added, and the sector imager was used to read the plate. We set a cutoff of 15% for intraassay coefficient of variation to repeat the assay. The overall coefficient of variation was 4.1%. We also repeated the measurement for each value >30 ng/mL, and each plate where three control CVs >2SD on the same plate, two control average >3SD on the same plate, two controls CVs >15% if the next plate CV >15% and one control CV >15% if the next plate >15%.
